# Pru p 3, a marker allergen for lipid transfer protein sensitization also in Central Europe

**DOI:** 10.1111/all.13151

**Published:** 2017-04-03

**Authors:** N. Mothes‐Luksch, M. Raith, G. Stingl, M. Focke‐Tejkl, E. Razzazi‐Fazeli, R. Zieglmayer, S. Wöhrl, I. Swoboda

**Affiliations:** ^1^ Comparative Immunology and Oncology Department of Pathophysiology and Allergy Research Center of Pathophysiology, Infectiology and Immunology Medical University of Vienna Vienna Austria; ^2^ Molecular Biotechnology Section FH Campus Wien University of Applied Sciences, Campus Vienna Biocenter Vienna Austria; ^3^ Department of Dermatology Division of Immunology, Allergy and Infectious Diseases Medical University of Vienna Vienna Austria; ^4^ Division of Immunopathology Department of Pathophysiology and Allergy Research Center for Pathophysiology, Infectiology and Immunology Medical University of Vienna Vienna Austria; ^5^ VetCore Facility for Research University of Veterinary Medicine Vienna Vienna Austria; ^6^ Vienna Challenge Chamber Vienna Austria; ^7^ FAZ ‐ Floridsdorf Allergy Center Vienna Austria

**Keywords:** component resolved diagnosis, food allergy, immunologic tests, lipid transfer protein, Pru p 3

## Abstract

In the Mediterranean area, lipid transfer proteins (LTPs) are important causes of plant‐food allergies often associated with severe allergic reactions. There, peach LTP (Pru p 3) seems to be the primary sensitizer, whereas in Central Europe, little is known about the importance of LTP sensitization. In this region, allergen extract‐based diagnosis is often complicated by co‐sensitization to Bet v 1, the major birch pollen allergen, its cross‐reactive food allergens, and profilins. We investigated the role of LTP sensitization in Central European patients displaying strong allergic reactions to plant‐derived food. Analysis of IgE reactivity revealed that ten of thirteen patients were sensitized to Pru p 3, nine to Bet v 1, and two to profilin. Our results showed that LTP sensitization represents a risk factor for severe allergic symptoms in Central Europe. Furthermore, the strong IgE reactivity detected in immunoblots of plant‐food extracts indicated that Pru p 3 can be used as a marker allergen for LTP sensitization also in Central European patients.

## 1 Introduction

Nonspecific lipid transfer proteins (nsLTPs) are extremely stable, structurally highly conserved plant defense proteins, present throughout the whole plant kingdom.^1^, ^2^, ^3^ LTPs have also been identified as important, cross‐reactive plant‐food allergens in fruits, vegetables, nuts, and cereals (reviewed in 4 and 5). Sensitization to LTPs is known to occur frequently in individuals living in the Mediterranean area, where it is often associated with severe, sometimes life‐threatening reactions.^5^, ^6^, ^7^, ^8^ Peach LTP (Pru p 3) represents the molecule that dominates the immune response to LTPs in these countries and is regarded as a marker for severe systemic reactions to plant‐derived food.^5^ Awareness of LTP sensitization is also increasing in Northern and Central Europe.^9^, ^10^, ^11^, ^12^ There, diagnosis of LTP sensitization using plant extracts is difficult due to a frequent co‐sensitization to birch pollen allergens, especially the major birch pollen allergen Bet v 1, Bet v 1 cross‐reactive food allergens, and profilins, all heat labile proteins, known as inducers of mild clinical manifestations.^5^ We therefore investigated whether LTPs also play a major role in Central European patients with severe reactions to plant‐derived foods and whether Pru p 3 can also serve as a diagnostic cross‐reactive marker allergen in patients in this region.

## 2 Materials and Methods

Thirteen Austrian patients with a clinical history of either severe anaphylactic reactions to plant‐food material or strong SPT reactions of ≥8 mm wheal diameter^13^ to a broad range of plant material were selected (Table S1). Ethical approval and written consent were obtained from the Austrian ethics committee (EK Nr: 1052/2013). Skin tests and determination of total and allergen‐specific IgE were carried out as described in Methods S1 and S2.

Plant extract preparation is described in Method S3. For immunoblotting, 10 μg of raw and cooked protein extracts of parsley, apricot peel, peach peel, and raspberry was separated by 15% Tricine SDS‐PAGE and transferred to nitrocellulose membranes (1 hour, 100 Volt) using a CAPS‐based transfer buffer (10 mmol/L CAPS, 10% methanol). For identification of LTPs, membranes were incubated with a rabbit anti‐Pru p 3 antiserum (1:10 000 dilution) as described in 14, a kind gift from ALK‐Abelló, Madrid, Spain. For the identification of Bet v 1 cross‐reactive proteins, membranes were incubated with a polyclonal rabbit antiserum directed against Bet v 1 (1:5000 dilution) as described in 15. Antibody binding was detected with HRPO‐labeled goat anti‐rabbit antibodies (0.1 μg/mL, Vector Laboratories, Burlingame, CA). For determination of IgE reactivity, membranes were incubated with individual patients' sera (1:10 dilution) and detected with HRPO‐labeled anti‐human IgE antibody (0.5 μg/mL Southern Biotech, Birmingham, AL).

For mass spectrometry analysis, gel slices were excised from the appropriate region (LTP~12 kDa) of Coomassie‐stained 15% Tricine SDS‐PAGE gels and subjected to in‐gel tryptic digestion (Thermo Fisher Scientific, Waltham, MA). Protein identification was performed as described by Hemmer et al.^16^ for the 12‐kDa bands of parsley, apricot, and peach and as described by Enk et al.^17^ for the raspberry band. The peak lists were cross‐referenced against the databases UniProt and Swiss‐Prot.

ImmunoCAP IgE‐inhibition experiments were carried out as described in Method S4.

## 3 Results

The patients enrolled in this study either experienced severe reactions upon ingestion of plant‐derived food or showed strong reactivity to various plant extracts in skin prick tests (for details, see Table S1). To assess the patients' reactivity profiles by single component‐resolved diagnosis (CRD), specific IgEs to rPru p 3, rBet v 1, and rPhl p 12 (the profilin from *Phleum pratense*) were determined by ImmunoCAP, ImmunoCAP ISAC, and/or ELISA. Interestingly, ten of the patients had IgE antibodies to Pru p 3 (76.9%), nine (69.2%) to Bet v 1, and only two (15.4%) to profilin (Table 1). These findings are consistent with the high prevalence of birch pollinosis in Central Europe, but they further indicate that LTPs might also cause severe symptoms in Central European population. The importance of CRD for determination of plant‐derived food allergy is underlined by our finding that the majority of the Pru p 3‐positive patients were also positive for Bet v 1 (60%), and consistent with the recommendations of the EAACI for food allergy and anaphylaxis.^18^

**Table 1:** Measurement of specific IgEs to rPru p 3, rBet v 1, or rPhl p 12

Subject	ImmunoCAP	ISAC microarray	ELISA	
	rPru p 3 (kU/L)	rPru p 3	rBet v 1	rPhl p 12
1	2.20	n.d.	pos	pos
2	4.08	n.d.	neg	neg
3	0.00	n.d.	pos	neg
4	0.92	n.d.	neg	neg
5	6.12	n.d.	pos	neg
6	0.00	n.d.	pos	neg
7	0.54	n.d.	pos	pos
8	12.70	n.d.	pos	neg
9	2.64	n.d.	pos	neg
10	3.79	n.d.	neg	neg
11	n.d.	neg	pos	neg
12	n.d.	pos	pos	neg
13	n.d.	pos	neg	neg

We went on to establish whether Pru p 3 is an appropriate marker protein for the diagnosis of LTP sensitization in Central Europe. Therefore, we determined patients' IgE reactivity to raw and cooked extracts from peach and from other plant foods which are an integral part of the Austrian cuisine, such as parsley, raspberry, and apricot, a fruit closely related to peach, but more popular in Austria. First, immunoblots performed with an antiserum against Pru p 3 clearly indicated the presence of LTPs in all extracts with the exception of raw parsley extracts. The results also indicate that cooking did not influence the recognition of the heat‐stable LTPs (Fig. S1A). Mass spectrometry analysis allowed the detection of LTP also in raw and cooked parsley extracts (Table S2). In contrast, an anti‐Bet v 1 antiserum detected Bet v 1‐related food allergens in immunoblots only in raw apricot and peach. Antibody binding to the Bet v 1‐related proteins was clearly diminished or even lost in the cooked extracts, suggesting that heat treatment denatured the Bet v 1‐related proteins (Fig. S1B).

**Figure 1:**
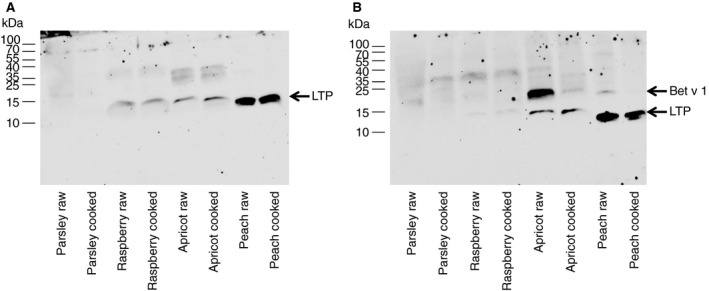
Representative immunoblots showing the IgE‐reactivity patterns of patients to proteins from plant extracts. Raw or cooked plant extracts were separated by 15% Tricine SDS‐PAGE, blotted onto nitrocellulose and exposed to the serum (A) of a LTP‐sensitized patient (patient 2) or (B) of a patient (patient 5) sensitized to both, LTP and Bet v 1. Molecular weight markers are shown on the left side of each blot

Based on the IgE‐reactivity patterns observed in the immunoblots, patients fell into two main groups: group 1 patients monosensitized to LTP and group 2 patients sensitized to both, LTP and Bet v 1. Group 1 (patients 2, 4, 10, and 13) showed bands at the molecular weight corresponding to LTP in the extracts of raspberry, apricot, and peach (a representative immunoblot of patient 2 is shown in Figure 1A). As expected, raw and cooked extracts were recognized to the same extent. Interestingly, the peach extract showed the strongest IgE reactivity. In group 2 (patients 1,5,7,8,9, and 12) reactivity to raspberry, apricot, and peach was observed at the molecular weight corresponding to LTP in raw and cooked extracts (a representative blot of patient 5 is shown in Figure 1B). In contrast, bands at the molecular weight corresponding to Bet v 1 were only visible in the raw extracts of apricot and peach and to a minor extent in the cooked extract of apricot (Figure 1B). These results indicate the following:

1. there is a benefit of using cooked extracts for prick to prick tests in the diagnosis of LTP sensitization as also suggested by Asero et al.^19^ and
2. in all of our ten LTP‐sensitized patients, raw and cooked extracts from peach showed strongest IgE reactivity, suggesting that Pru p 3 carries most of the epitopes recognized by patients' IgEs.

Even though the primary LTP‐sensitizing source is also not always known in Mediterranean countries, the majority of LTP‐sensitized patients in this area react with Pru p 3 and Pru p 3 usually initiates the LTP‐allergy syndrome.^5^ In contrast, the LTP‐sensitizing source in Central Europe still remains totally elusive. We therefore performed ImmunoCAP inhibition experiments with those two cooked extracts that had shown strongest IgE reactivity in the IgE immunoblots, namely apricot and peach. Both extracts inhibited IgE reactivity to rPru p 3 (Table S3) and to the peach and apricot ImmunoCAPs (data not shown) in a similar way, most likely due to the high sequence homology between LTPs from peach and apricot. Thus, it is still possible that apricot rather than peach is the source for LTP sensitization also in Central Europe. However, due to the close phylogenetic relationship between peach and apricot, the primary sensitizer could not be identified unequivocally.

## 4 Discussion

We have established that nsLTPs play an important role as plant‐food allergens in a Central European population reacting with strong symptoms. Patients from the central and northern parts of Europe, where primary sensitization to Bet v 1 usually occurs via the respiratory tract, often present only with mild symptoms after consumption of plant‐food material. In these subjects, plant‐derived food allergy is mediated by Bet v 1‐related proteins causing allergic cross‐reactions due to the high sequence similarity with Bet v 1. In contrast, allergic reactions to plant‐derived food in Southern Europe are usually associated with systemic reactions, due to a primary sensitization to LTPs, very potent allergens, usually causing severe reactions.

However, severe reactions to fruits, vegetable, and storage plants do also occur in Northern and Central European individuals. This demands a careful, diagnostic workup scheme for allergic patients, which fulfills the needs to distinguish between different highly cross‐reactive allergens. Bet v 1‐related allergens, profilins, and the nsLTPs are widely distributed throughout the plant kingdom, limiting the use of crude plant extracts for differential diagnostic purposes.

A major drawback of CRD is that the clinical relevance of the marker allergens requires a clinical validation and marker allergens should be recognized by the majority of the patients in each geographical area. As all Austrian LTP‐sensitized patients screened for specific IgEs to peach and to plants representative of a Central European Cuisine displayed strongest IgE reactivity to peach extract, we conclude that Pru p 3 can be used as the marker allergen for LTP sensitization also in Central Europe.

The fact that extracts from peach and from apricot inhibited IgE reactivity to rPru p 3 similarly showed that due to the high sequence similarity between LTPs within the *Rosaceae* family, it might be impossible to identify the LTP‐sensitizing source in Central Europe.

## Conflicts of interest

The authors declare no conflict of interests regarding the publication of this article.

## Author contributions

N. Mothes‐Luksch, M. Raith, G. Stingl, S. Wöhrl, and I. Swoboda designed the research. N. Mothes‐Luksch, M. Raith, M. Focke‐Tejkl, E. Razzazi‐Fazeli, and R. Zieglmayer performed the experiments. N. Mothes‐Luksch, M. Raith, S. Wöhrl, and I. Swoboda wrote the manuscript. All authors read and approved the final manuscript.
