# Supplementary material for: Pru p 3, a marker allergen for lipid transfer protein sensitization also in Central Europe
Source: Allergy. 2017 Apr 3;72(9):1415–8. doi: 10.1111/all.13151 (PMC5573991; doi:10.1111/all.13151)
Supplement: Supplementary file 1 [file ALL-72-1415-s001.docx]

**Supplementary Figure 1**

**Supplementary Figure 1. Detection of Pru p 3 and Bet v 1-related proteins in raw and cooked plant extracts.** Raw and cooked extracts from parsley (*Petroselinum crispum*), raspberry (*Rubus idaeus*), apricot (*Prunus armeniaca*), and peach (*Prunus persica*) were separated on 15% Tricine SDS-PAGEs and blotted onto nitrocellulose. Membranes were incubated with antisera directed against Pru p 3 (**A**) or Bet v 1 (**B**). Protein markers are shown on the left margins.

Raspberry raw

Parsley cooked

Raspberry cooked

Parsley raw

Apricot raw

Peach raw

Peach cooked

Apricot cooked

10

15

25

35

40

55

100

70

70

10

15

25

35

40

55

100

A

B

kDa

kDa

**anti-Pru p 3**

**anti-Bet v 1**


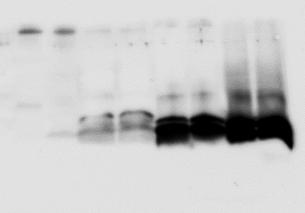

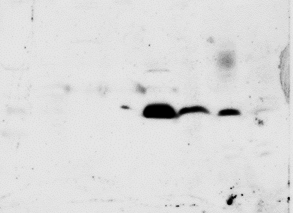


Raspberry raw

Parsley cooked

Raspberry cooked

Parsley raw

Apricot raw

Peach raw

Peach cooked

Apricot cooked

**Supplementary Figure 2. Protein patterns of raw and cooked plant extracts.** Raw or cooked extracts from parsley (*Petroselinum crispum*), raspberry (*Rubus idaeus*), apricot (*Prunus armeniaca*), and peach (*Prunus persica*) were loaded on a 15% Tricine SDS-PAGE and subsequently stained with Coomassie dye. A protein marker is shown on the left margin. Equal volumes of the raw and cooked extracts were always loaded on the gel.

**Supplementary Figure 2**


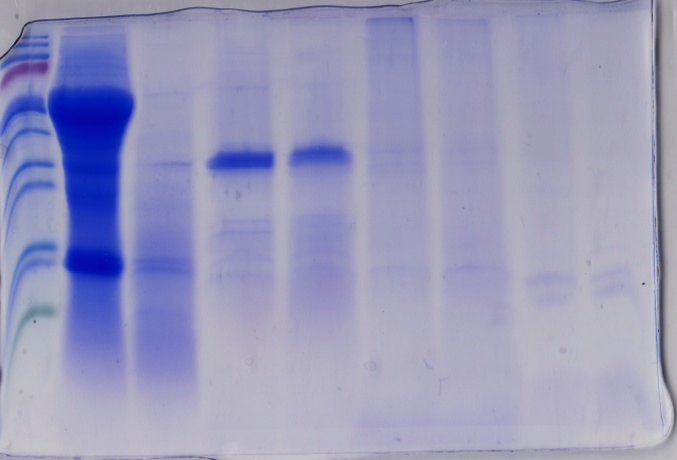


10

15

25

35

40

55

130

100

70

170

Marker

kDa

Raspberry raw

Parsley cooked

Raspberry cooked

Parsley raw

Apricot raw

Peach raw

Peach cooked

Apricot cooked
