# Supplementary material for: Pru p 3, a marker allergen for lipid transfer protein sensitization also in Central Europe
Source: Allergy. 2017 Apr 3;72(9):1415–8. doi: 10.1111/all.13151 (PMC5573991; doi:10.1111/all.13151)
Supplement: Supplementary file 2 [file ALL-72-1415-s002.docx]

**Supplementary Table 1.** Demographic and clinical characterization of patients

| **Subject** | **Age/Sex** | **Total IgE (kU/L)** | **Symptoms** | **positive SPT*** |
| --- | --- | --- | --- | --- |
| 1 | 34/M | >5,000 | no symptoms after ingestion of foods  atopic dermatitis | parsley, hazelnut, peanut, strawberry, blackberry, birch, grasses, mugwort, Alternaria, peach SPT solution** |
| 2 | 56/F | 45.4 | anaphylactic reaction (Mueller grade III) after ingestion of parsley and other plant foods, OAS after raspberries | peach, blackberry, buckshorn, mites, ash, peach SPT solution** |
| 3 | 59/F | 639 | exercise induced anaphylactic reaction (Mueller grade III) after ingestion of banana, grapes and hazelnuts | parsley, hazelnut, blackberry, birch, peach SPT solution** |
| 4 | 62/F | 163 | nausea, pruritus (Mueller grade II-III) after ingestion of peach, apple, raspberry and plum, avoids nuts for years | peach, apple, birch, mugwort, mites, peach and date SPT solutions** |
| 5 | 28/F | 284 | anaphylactic reaction (Mueller grade III-IV) after fruit salad including peach, apples and melon, raspberry and strawberry, avoids nuts for years | hazelnut, strawberry, raspberry, blackberry, peach, apple, birch, grasses, Alternaria, mites, animal dander, peach and date SPT solutions** |
| 6 | 44/M | 558 | 4 times anaphylactic reaction (Mueller grade III) after ingestion of pizza, apple, raspberry and black currant juice | hazelnut, birch |
| 7 | 43/F | 763 | atopic dermatitis since childhood, allergic rhino-conjunctivitis, only birch-related oral allergy syndrome | parsley, hazelnut, peanut, strawberry, raspberry, blackberry, profilin, birch, date SPT solution** |
| 8 | 22/F | 246 | exercise induced dyspnea and urticaria (Mueller grade II) after ingestion of peach and hazelnut | parsley, hazelnut, peanut, raspberry, blackberry, birch, grasses, mugwort, ragweed, cat dander, dog dander, peach SPT solution** |
| 9 | 44/M | 215 | anaphylactic reaction (Mueller grade II) of unknown cause in connection with NSAR intake | parsley, hazelnut, peanut, strawberry, raspberry, blackberry, peach, apple, birch, grasses, mites, peach SPT solution** |
| 10 | 64/M | 154 | anaphylactic reaction (Mueller grade II) after ingestion of nuts and fruit desert with kiwi and berries | parsley, hazelnut, peanut, strawberry, raspberry, blackberry, peach, grasses, olive tree, plane tree, peach SPT solution** |
| 11 | 18/F | 804 | face erythema after ingestion of peach | birch, grasses, weed, mites, dander |
| 12 | 24/M | >5,000 | birch-related oral allergy syndrome | birch, grasses |
| 13 | 60/M | 155 | exercise-induced hypotension, pruritus, vision disorders (Mueller grade III) after ingestion of apple strudel, sauerkraut and juniper berry | parsley, raspberry, peach |

* allergen sources and allergens resulting in a positive skin reaction

** positive reactions to skin prick test solutions (SPT) of peach (enriched in Pru p 3) and date (enriched in
profilin)

**Supplementary Table 2. Identification of LTPs in plant-derived food by Nano LC-ESI MS/MS**.

| **Sample** | **Identified LTP** | | **number of identified peptides** |
| --- | --- | --- | --- |
|  | **Accession number** | **species** |  |
| Parsley | P27056 | *Solanum lycopersicum* | 1 |
| Raspberry | Q0Z8V0 | *Rubus idaeus* | 11 |
| Apricot | P81651 | *Prunus armeniaca* | 4 |
| Peach | P81402 | *Prunus persica* | 3 |

**Supplementary Table 3. Percentages of inhibition of IgE-reactivity to rPru p 3 after preadsorption of 5 patients’ sera (2, 4, 8, 9, 10) with cooked peach or apricot extract as determined by ImmunoCAP analysis.**

| **Patient** | **% of inhibition** | |
| --- | --- | --- |
|  | **peach extract** | **apricot extract** |
| **2** | 93 | 88 |
| **4** | 62 | 66 |
| **8** | 82 | 83 |
| **9** | 73 | 78 |
| **10** | 62 | 65 |
