# Supplementary material for: Pru p 3, a marker allergen for lipid transfer protein sensitization also in Central Europe
Source: Allergy. 2017 Apr 3;72(9):1415–8. doi: 10.1111/all.13151 (PMC5573991; doi:10.1111/all.13151)
Supplement: Supplementary file 3 [file ALL-72-1415-s003.docx]

**Supplemental Methods**

***S1*** *Skin testing*

For analysis of sensitization to plant-derived food, prick tests were performed as prick-to-prick tests with fresh or cooked plant material. For analysis of sensitization to inhalant allergen sources, prick test were carried out with commercial skin prick test solutions (ALK-Abelló). To investigate sensitization to Pru p 3, a Pru p 3-enriched commercial peach skin prick test solution (ALK-Abelló) was used and to investigate profilin sensitization a profilin-enriched date skin prick test solution (ALK-Abelló) was used.

***S2*** *Determination of total and allergen-specific IgE*

Total IgE was measured by ImmunoCAP (Phadia-Thermo Fisher Scientific, Uppsala, Sweden). Determination of allergen-specific IgEs was carried out by ImmunoCAP or ImmunoCAP ISAC (both Phadia-Thermo Fisher Scientific) or by ELISA. For the determination of anti-Bet v 1 or anti-Phl p 12 specific IgE antibodies, MaxiSorp flatbottom 96-well plates (Thermo Fisher Scientific, Waltham, MA) were coated with 0.4 µg/well rBet v 1 (Biomay, Vienna, Austria) or with 0.4 µg/well rPhl p 12 (produced as described by Valenta R. et al, except that there was an additional Histidin tag at the C-terminus) in bicarbonate buffer (37 mM Na_2_CO_3_, 63 mM NaHCO_3_, pH 9.6) overnight. Plates were washed 5 times with TBS-T (10 mM Tris-HCl with a pH 8.0, 150 mM NaCl, 0.5% Tween20) and blocked for 2.5 hours at 37°C with 1% BSA in TBS-T. Patients’ sera were 1:5 diluted in TBS-T, added to the wells and incubated overnight. After washing the plates 5 times with TBS-T, 0.5 µg/ml AP-labeled secondary antibodies to human IgE (Becton Dickinson, Franklin Lakes, NJ) were added and incubated for 1 hour at 37°C and another hour at 4°C. Wells were washed with TBS-T and incubated with phosphatase substrate (4-Nitrophenyl phosphatase disodium salt hexahydrate, Sigma Aldrich, St. Louis, MO) in ELISA substrate solution (2.6 mM NaN_3_, 712 mM diethanolamine, pH set to 9.8 with 1 N acetic acid) until an appropriate color reaction was visible. The OD of 405 nm, with a reference wavelength of 550 nm was measured in a Multiskan^TM^ plate reader (Thermo Fisher Scientific).

***S3*** *Preparation of plant extracts*

Fresh parsley (*Petroselinum crispum*), apricot (*Prunus persica*) and peach (*Prunus armeniaca*) and frozen raspberry (*Rubus idaeus*) were bought at the supermarket. For the aqueous extracts raspberry, parsley, apricot peel or peach peel were minced using a hand blender and 1 ml potassium phosphate buffer (10 mM) containing 3 mM sodium azide were added per gram plant material, except for the raspberry, where the salts were added directly to the fruit pulp to avoid a further dilution of the extract due to the high content of water. After extraction for 30 minutes at 4°C under constant stirring, the extract was centrifuged at 4,000 g for 20 minutes and the supernatant was further clarified by centrifugation for another 30 minutes at 23,400 g. To obtain cooked extracts, native extracts were boiled for 15 minutes at 100°C in a water bath. Extracts separated on a Coomassie-stained SDS-PAGE are shown in Supplementary Figure 2.

***S4*** *IgE inhibition experiments*

For the ImmunoCAP inhibition experiments, cooked extracts from peach and apricot, were used. Sera from 5 patients (each serum diluted 1:2 in PBS) were pre-incubated overnight at 4°C with either 5 µg of peach or apricot extracts or, for control purposes, with PBS. The remaining IgE-reactivity to Pru p 3 was measured using the ImmunoCAP (Phadia-ThermoFisher Scientific) system.

**Supplemental reference**

Valenta R, Ball T, Vrtala S, Duchene M, Scheiner O. cDNA cloning and expression of timothy grass (Phleum pratense) pollen profilin in Escherichia coli: comparison with birch pollen profilin. *Biochem Biophys Res Commun* 1994;199:106–118.
